# Supplementary material for: Reversion to sensitivity explains limited transmission of resistance in a hospital pathogen
Source: bioRxiv. 2024 Jun 3:2024.06.03.597162. Preprint. [Version 1] doi: 10.1101/2024.06.03.597162 (PMC11185621; doi:10.1101/2024.06.03.597162)
Supplement: Supplement 1 [file NIHPP2024.06.03.597162v1-supplement-1.pdf]

bioRxiv preprint doi: https://doi.org/10.1101/2024.06.03.597162; this version posted June 3, 2024. The copyright holder for this preprint (which was not certified by peer review) is the author/funder. This article is a US Government work. It is not subject to copyright under 17 USC 105 and is also made available for use under a CC0 license.

7 Supplemental Data and Figures

Patient	Phenotype	Group ID	MLST	Isolate Number	Accession	Clinical MIC (μg/mL)
DN1	Sensitive	DNS	412	BL00201-1	SAMN35347354	2
DN2	Sensitive	DNS	17	BL00216-1	SAMN35347358	4
DN3	Sensitive	DNS	18	BL00239-1	SAMN35347362	4
DN4	Sensitive	DNS	584	BL00242-1	SAMN35347366	4
DN5	Sensitive	DNS	17	BL00244-1	SAMN35347370	2
DN6	Sensitive	DNS	18	BL00247-1	SAMN35347374	4
DN1	Resistant	DNR	412	BL00211-1	SAMN35347378	8
DN2	Resistant	DNR	17	BL00223-1	SAMN35347382	32
DN3	Resistant	DNR	18	BL00241-1	SAMN35347386	8
DN4	Resistant	DNR	584	BL00243-1	SAMN35347390	8
DN5	Resistant	DNR	17	BL00246-1	SAMN35347394	16
DN6	Resistant	DNR	18	BL00250-1	SAMN35347398	16
PT1	Resistant	PTR	1471	BL00192-1	SAMN35347330	16
PT2	Resistant	PTR	-	BL00194-1	SAMN35347334	8
PT3	Resistant	PTR	1471	BL00196-1	SAMN35347338	8
PT4	Resistant	PTR	664	BL00198-1	SAMN35347342	8
PT5	Resistant	PTR	412	BL00184-1	SAMN35347346	8
PT6	Resistant	PTR	664	BL00777-1	SAMN35347350	4

Table 1: Associated metadata of the study isolates and their respective phenotype, MLST, study grouping, NCBI accession, and isolate number.

bioRxiv preprint doi: https://doi.org/10.1101/2024.06.03.597162; this version posted June 3, 2024. The copyright holder for this preprint (which was not certified by peer review) is the author/funder. This article is a US Government work. It is not subject to copyright under 17 USC 105 and is also made available for use under a CC0 license.

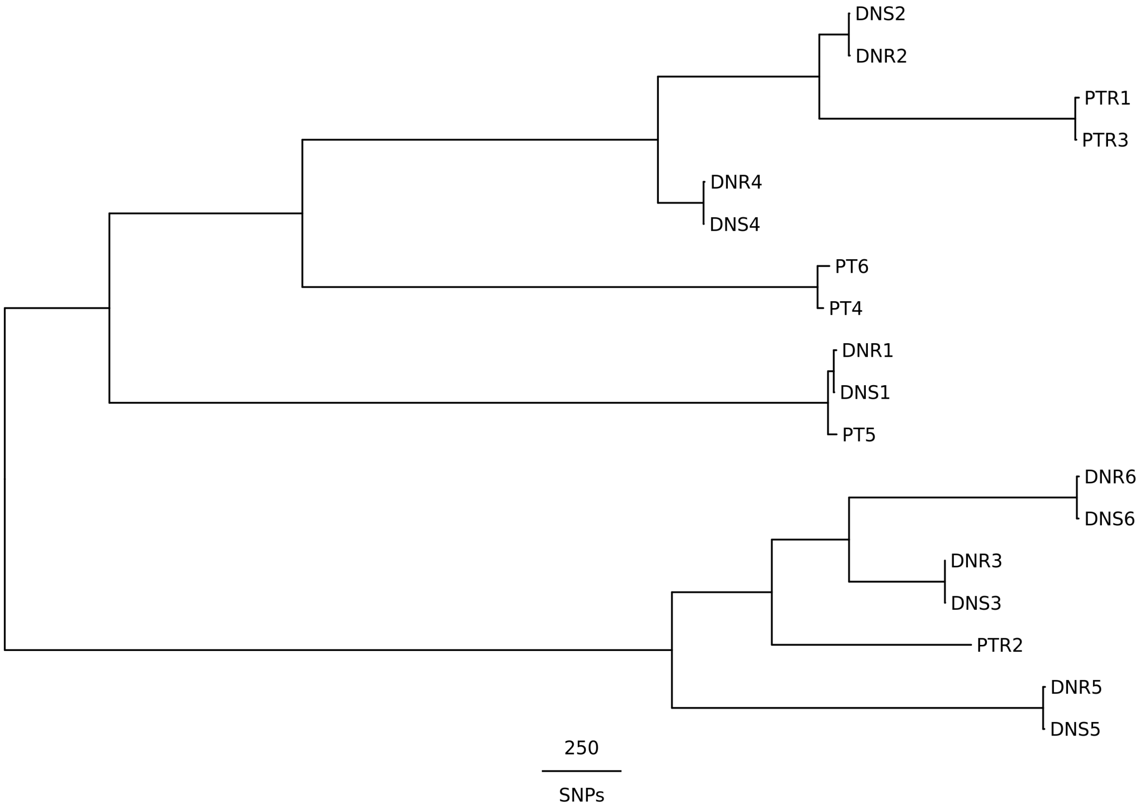

Figure 6: **Phylogenetic tree of the 18 initial, unevolved isolates** A neighbor-joining tree of the initial 18 founding isolates from the SNP distance matrix. The SNP distance matrix was derived from a core genome alignment of all 18 isolates.

bioRxiv preprint doi: https://doi.org/10.1101/2024.06.03.597162; this version posted June 3, 2024. The copyright holder for this preprint (which was not certified by peer review) is the author/funder. This article is a US Government work. It is not subject to copyright under 17 USC 105 and is also made available for use under a CC0 license.

Locus	Locus Tag (HMPREF0351)	Accession Number	direction	start	stop
Aad	10204	YP_006374810	+	202396	204996
TelA	10540	YP_006375146	−	536179	537375
DltA	10751	YP_006375357	+	755153	756667
DltB	10752	YP_006375358	+	756664	757869
DltC	10753	YP_006375359	+	757914	758147
DltD	10754	YP_006375360	+	758150	759418
LiaF	10936	YP_006375542	+	912994	913749
LiaS	10937	YP_006375543	+	913746	914813
LiaR	10938	YP_006375544	+	914820	915452
PTS−EIIA	11023	YP_006375629	+	1010361	1010822
Cls	11068	YP_006375674	−	1050806	1052287
MprF/FmtC	11082	YP_006375688	−	1067539	1070139
GdpD/GlpQ	11730	YP_006376336	+	1706101	1707900
PTS−IIA	11792	YP_006376398	−	1759099	1759509
HD Domain protein	11908	YP_006376514	−	1869447	1869950
PspC	12014	YP_006376620	−	1967889	1968209
YycJ	12358	YP_006376964	−	2303657	2304466
YycI	12359	YP_006376965	−	2304521	2305390
YycH	12360	YP_006376966	−	2305391	2306707
YycG/Walk/VicK	12361	YP_006376967	−	2306704	2308539
YycF/WalR/VicR	12362	YP_006376968	−	2308544	2309278

Table 2: Candidate daptomycin resistance genes as reported in Diaz et al (*38*) with mutations occurring across multiple MLSTs removed (*35*).

	Value	Std.Error	DF	t-value	p-value
**MODEL 1: $\log_2$(final MIC-initial MIC) ˜ Group**					
Intercept	-0.6999106	0.5890485	108	-1.19	0.24
Group	-1.0040332	0.8330403	10	-1.2052635	0.2558477
**MODEL 2: $\log_2$(final MIC-initial MIC) ˜ Group + $\log_2$(initial MIC)**					
Intercept	1.2206256	0.3132934	107	3.90	0.0002
Group	-0.2835307	0.3981069	10	-0.71	0.49
$\log_2$(initial MIC)	-1.0170222	0.0755229	107	-13.47	<0.001
**MODEL 3: MIC_log2 ˜ Group + InitMIC_log2 + Time**					
(Intercept)	1.8352468	0.2789396	143	6.58	<0.001
GroupDNR	-0.2057706	0.2972867	10	-0.69	0.50
InitMIC_log2	0.2275255	0.0634888	143	3.58	0.0004
Time	-0.0400642	0.0063254	107	-6.33	<0.001

Table 3: Maximum likelihood fit of three mixed models, as described in the text, to estimate the effect of group (*i.e.* being founded with a PT or DN clone), and the MIC of that founding clone, on the reduction in MIC after 32 days of evolution in antibiotic-free conditions. difference (individual) as output, does NOT include DNS group

bioRxiv preprint doi: https://doi.org/10.1101/2024.06.03.597162; this version posted June 3, 2024. The copyright holder for this preprint (which was not certified by peer review) is the author/funder. This article is a US Government work. It is not subject to copyright under 17 USC 105 and is also made available for use under a CC0 license.

	Value	Std.Error	DF	t-value	p-value
**MODEL 1: Fitness ˜ Group(DNR/DNS)**					
(Intercept)	0.7119821	0.13758223	17	7.99	<0.001
GroupDNS	-0.1600093	08064874	10	-1.98	0.0636
**MODEL 2: Fitness ˜ Group (DNR/PTR)**					
(Intercept)	1.2158757	0.2063990	24	5.89	<0.001
GroupDNR	-0.1171131	0.2918923	10	-0.40	0.6967
**MODEL 3: Fitness ˜ Day**					
(Intercept)	1.0844638	0.11930478	108	9.09	<0.001
Day 32	0.6258661	0.08279977	53	7.56	<0.001
**MODEL 4: Fitness difference ˜ Initial MIC**					
(Intercept)	1.5714047	0.2813577	72	5.59	<0.001
Day 0 fitness	-0.8548231	0.1897687	23	-4.50	2e-04

Table 4: Maximum likelihood fit of mixed models to estimate the effect of group (*i.e.* being founded with a PT or DN clone), fitness gain during adaptation, and starting MIC on the fitness of strains after 32 days of evolution in antibiotic-free conditions.

Competition Model

We first fit the single species logistic growth model with a lag phase (*21*), where $\alpha(t) = \frac{q}{q+e^{-mt}}$. Parameters were estimated on each of the three clones taken from the 18 experimental population (6 DNR, 6 DNS, 6 PTR) across three biological replicates. Models were fit using POMP (*23*) with the Nelder-Mead optimization using the optim package. Models were fit assuming log-normal errors.

$$\frac{dN}{dt} = \alpha(t)rN(1 - \frac{N}{K}) \tag{2}$$

The parameter estimates taken from the single species model are then used to estimate the relative fitness values using the single resource competition model derived by Ram et al 2019 (*22*). Briefly summarizing, let R be the density of the limiting resource and N be density of cell populations. Cell growth is assumed to be proportional to R·N, resource is taken up by cells at rate h, and converted to cell mass at ϵ

bioRxiv preprint doi: https://doi.org/10.1101/2024.06.03.597162; this version posted June 3, 2024. The copyright holder for this preprint (which was not certified by peer review) is the author/funder. This article is a US Government work. It is not subject to copyright under 17 USC 105 and is also made available for use under a CC0 license.

$$\frac{dR}{dt} = -h_1RN_1 - h_2RN_2 \tag{3a}$$

$$\frac{dN_1}{dt} = \epsilon_1 h_1 RN_1 \tag{3b}$$

$$\frac{dN_2}{dt} = \epsilon_2 h_2 RN_2 \tag{3c}$$

By conservation of mass:

$$M_i = \epsilon_i R + N_i + \frac{\epsilon_i}{\epsilon_j} N_j \tag{4}$$

Since $\frac{dM_i}{dt} \equiv 0$ and M_i is constant

$$\epsilon_i R = M_i - N_i - \frac{\epsilon_i}{\epsilon_j} N_j \tag{5}$$

Substituting 5 into 3b and 3c, we get

$$\frac{dN_1}{dt} = h_1 N_1 (M_1 - N_1 - \frac{\epsilon_1}{\epsilon_2} N_2)) \tag{6a}$$

$$\frac{dN_2}{dt} = h_2 N_2 (M_2 - N_2 - \frac{\epsilon_2}{\epsilon_1} N_1))) \tag{6b}$$

Let $K_i = M_i$, $c_1 = \frac{\epsilon_2}{\epsilon_1}$, $c_2 = \frac{\epsilon_1}{\epsilon_2}$, $h_i K_i = r$

$$\frac{dN_1}{dt} = r_1 N_1 (1 - \frac{N_1}{K_1} - c_2 \frac{N_2}{K_1}) \tag{7a}$$

$$\frac{dN_2}{dt} = r_2 N_2 (1 - \frac{N_2}{K_2} - c_1 \frac{N_1}{K_2}) \tag{7b}$$

From the single species logistic growth:

$$K_i = \epsilon_i R + N_i \tag{8}$$

Ram et al assumes that $c_1 = c_2 = 1$ is an appropriate approximation based on empirical data. This assumption causes bacterial populations to decline if K is significantly different between

bioRxiv preprint doi: https://doi.org/10.1101/2024.06.03.597162; this version posted June 3, 2024. The copyright holder for this preprint (which was not certified by peer review) is the author/funder. This article is a US Government work. It is not subject to copyright under 17 USC 105 and is also made available for use under a CC0 license.

the two species. This also implies that differences in R are driving the difference between K_1 and K_2, rather than a biological process

Rather we assume that the resources between the two single species systems are equal

$$\frac{K_1 - N_1}{\epsilon_1} = \frac{K_2 - N_2}{\epsilon_2} = R \tag{9}$$

We can simplify this expression by assuming

$$\frac{N_1}{\epsilon_1} = \frac{N_2}{\epsilon_2} \tag{10}$$

This provides the approximation

$$\frac{K_1}{\epsilon_1} \approx \frac{K_2}{\epsilon_2} = R \tag{11}$$

And we can then calculate c_1 and c_2 in terms of K_1 and K_2

$$\frac{K_1}{K_2} = \frac{\epsilon_1}{\epsilon_2} = c_2 \tag{12a}$$

$$\frac{K_2}{K_1} = \frac{\epsilon_2}{\epsilon_1} = c_1 \tag{12b}$$

We then add the lag phase term to the equation to give us

$$\frac{dN_1}{dt} = \alpha(t)r_1N_1(1 - \frac{N_1}{K_1} - c_2\frac{N_2}{K_1}) \tag{13a}$$

$$\frac{dN_2}{dt} = \alpha(t)r_2N_2(1 - \frac{N_2}{K_2} - c_1\frac{N_1}{K_2}) \tag{13b}$$

$$\frac{dN_1}{dt} = \alpha(t)r_1N_1(1 - \frac{N_1}{K_1} - \frac{N_2}{K_2}) \tag{14a}$$

$$\frac{dN_2}{dt} = \alpha(t)r_2N_2(1 - \frac{N_1}{K_1} - \frac{N_2}{K_2}) \tag{14b}$$

The relative fitness is calculated using:

$$W = \frac{\log \frac{N_1(20)}{N_1(0)}}{\log \frac{N_2(20)}{N_2(0)}} \tag{15}$$

bioRxiv preprint doi: https://doi.org/10.1101/2024.06.03.597162; this version posted June 3, 2024. The copyright holder for this preprint (which was not certified by peer review) is the author/funder. This article is a US Government work. It is not subject to copyright under 17 USC 105 and is also made available for use under a CC0 license.

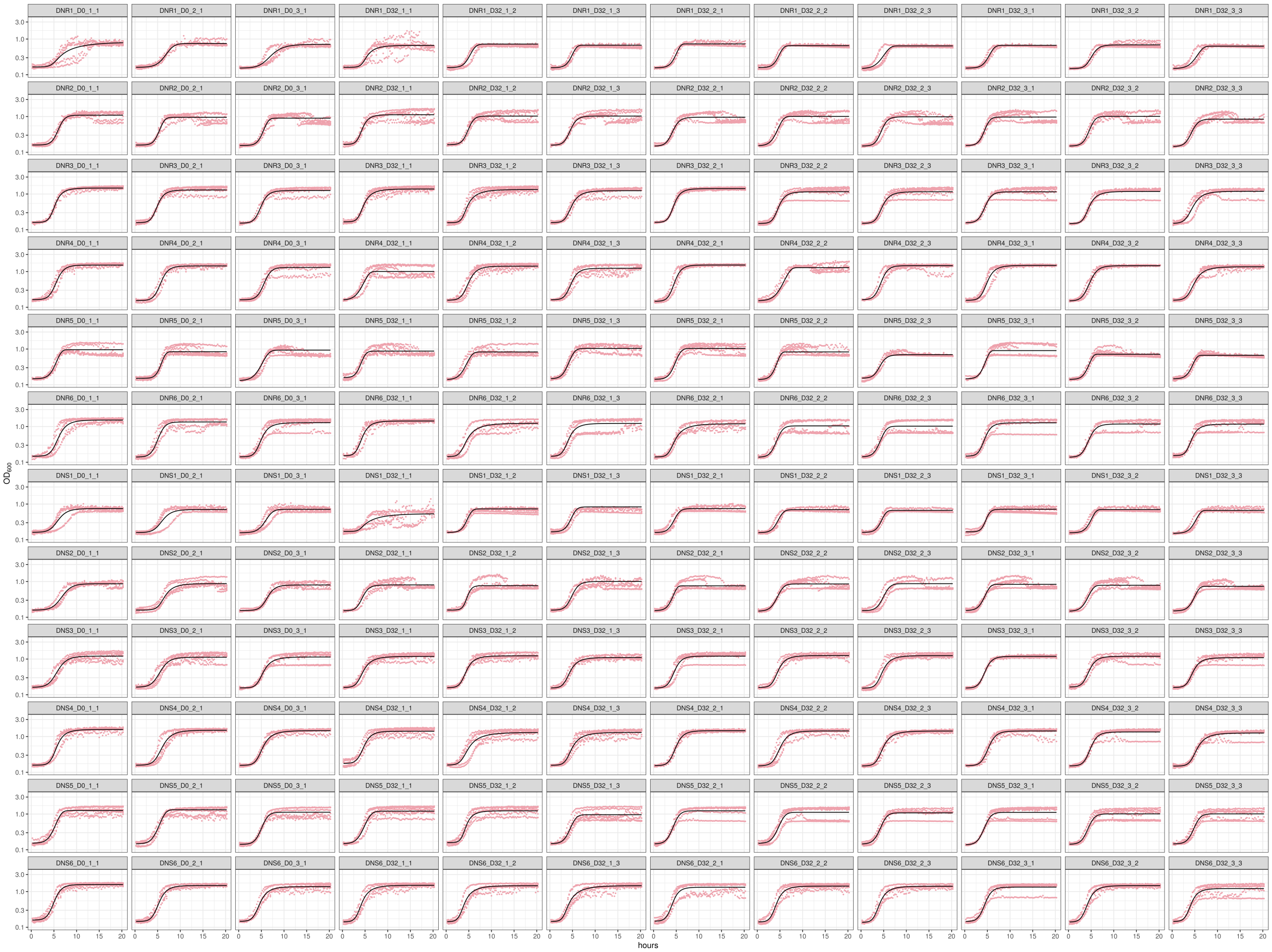

Figure 7: **Growth curves of *de novo* resistant strains** Each point represents one of 2 technical replicates from one of the three different biological replicates. The best fit from the growth model is superimposed. Each plot is labeled by Strain ID, Day, Experimental Population, and Clone

bioRxiv preprint doi: https://doi.org/10.1101/2024.06.03.597162; this version posted June 3, 2024. The copyright holder for this preprint (which was not certified by peer review) is the author/funder. This article is a US Government work. It is not subject to copyright under 17 USC 105 and is also made available for use under a CC0 license.

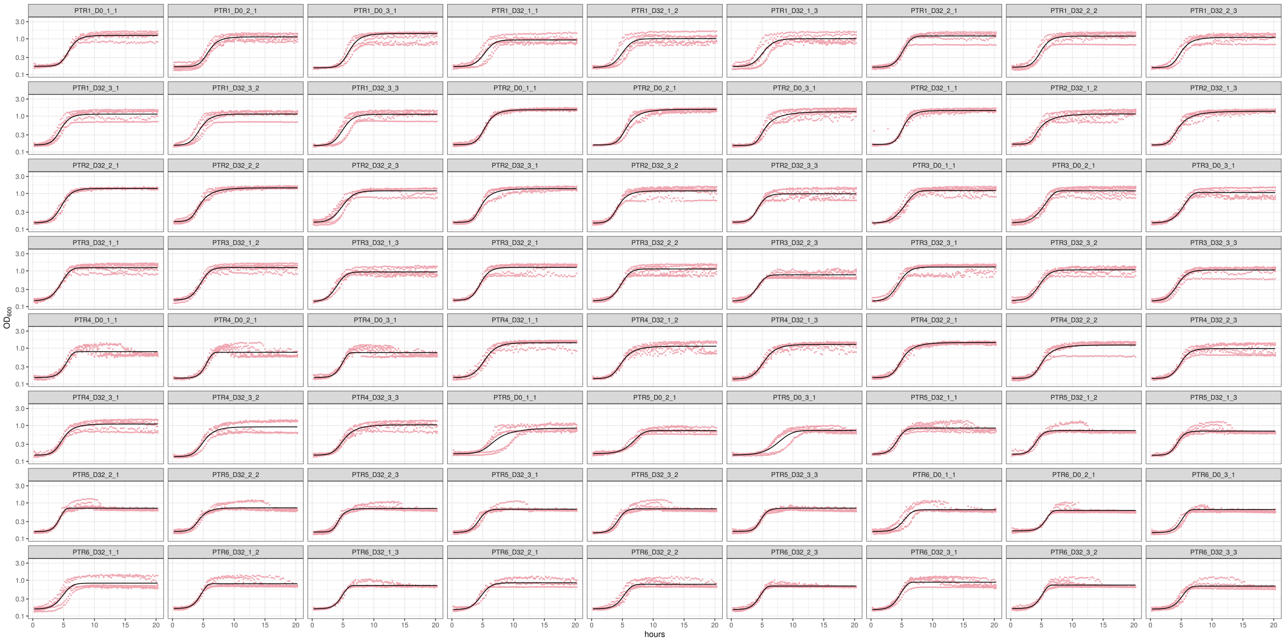

Figure 8: **Growth curves of transmitted resistant strains** Each point represents one of 2 technical replicates from one of the three biological replicates. The best fit from the model is superimposed. Each plot is labeled by Strain ID, Day, Experimental Population, and Clone

bioRxiv preprint doi: https://doi.org/10.1101/2024.06.03.597162; this version posted June 3, 2024. The copyright holder for this preprint (which was not certified by peer review) is the author/funder. This article is a US Government work. It is not subject to copyright under 17 USC 105 and is also made available for use under a CC0 license.

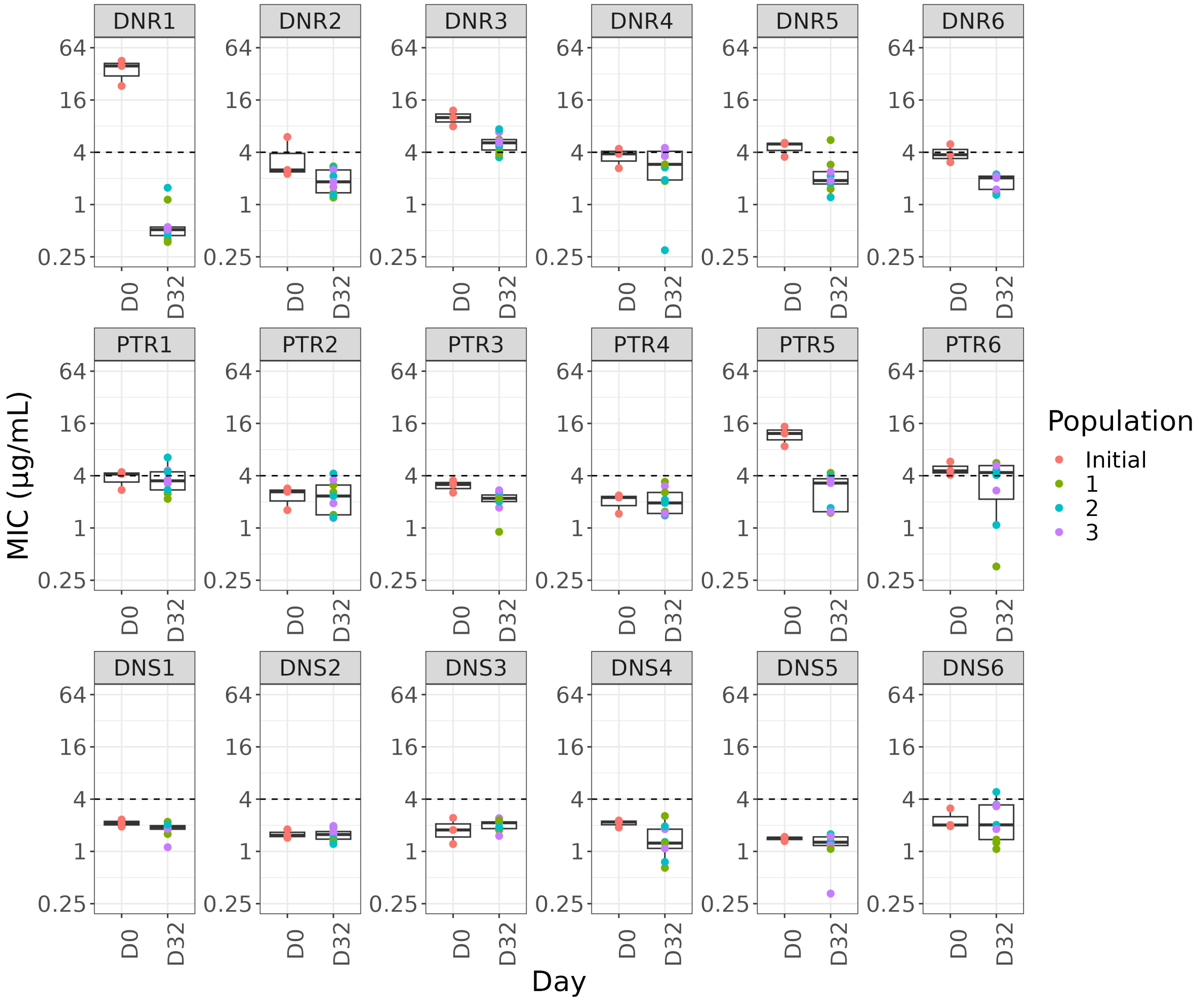

Figure 9: **Daptomycin resistance levels before and after evolution in an antibiotic-free environment** Minimum inhibitory concentration of daptomycin from clinical isolates (initial) and three isolates from each of three replicate populations following 320 generations of experimental evolution.
